# Intracytoplasmic sperm injection versus conventional in vitro insemination in couples with non-male infertility factor in the ‘real-world’ setting: analysis of the HFEA registry

**DOI:** 10.1186/s12967-024-05515-x

**Published:** 2024-07-28

**Authors:** Alessio Paffoni, Amerigo Vitagliano, Laura Corti, Edgardo Somigliana, Paola Viganò

**Affiliations:** 1 Infertility Unit, ASST Lariana, Cantù, Italy; 2 First Unit of Obstetrics and Gynecology, Department of Interdisciplinary Medicine, University of Bari, Bari, Italy; 3 Infertility Unit, Fondazione IRCCS Ca’ Granda Ospedale Maggiore Policlinico, Milan, Italy; 4 Department of Clinical Sciences and Community Health, Università Degli Studi di Milano, Milan, Italy; 5 Infertility Unit, Fondazione IRCCS Ca’ Granda Ospedale Maggiore Policlinico, Via F. Sforza, 36, 20122 Milan, Italy

**Keywords:** IVF, ICSI, Assisted reproductive technologies, HFEA, Conventional in vitro insemination

## Abstract

**Background:**

In assisted reproductive technology (ART), the choice between intracytoplasmic sperm injection (ICSI) and conventional in vitro insemination (IVF) remains a pivotal decision for couples with female or unexplained infertility. The hypothesis that ICSI may not confer significant improvements in live birth rates in the absence of a male infertility factor was explored in this study.

**Methods:**

This was a retrospective collection of data recorded by the Human Fertilisation and Embryology Authority (HFEA) in the UK from 2005 to 2018 and analysed through regression analysis models on both the entire dataset and a matched-pair subset. First fresh ART cycles were analysed according to the insemination technique in order to compare live birth as the main outcome. Cycles were included if complete information regarding infertility cause, female age, number of oocytes retrieved, allocation to ICSI or IVF, and treatment outcome in terms of live birth was available. Matching was performed at a 1:1 ratio between IVF and ICSI cycles according to the cause of infertility, female age, number of oocytes, and year of treatment.

**Results:**

This study, based on 275,825 first cycles, revealed that, compared with IVF, ICSI was associated with higher fertilization rates and lower cycle cancellations rates. However, ICSI was associated with a lower chance of implantation and live birth than IVF in cycles with female-only infertility: in the entire dataset, the adjusted odds of having a live birth decreased by a factor of 0.95 (95% CI 0.91–0.99, p = 0.011), while in the matched-pair analyses it decreased by a factor of 0.91 (95% CI 0.86–0.96, p = 0.003) using ICSI compared to IVF. For unexplained infertility cycles, the adjusted odds ratios for live birth in ICSI compared to IVF cycles were 0.98 (95% CI 0.95–1.01) in the entire dataset and 0.97 (95% CI 0.93–1.01) in the matched-pair analysis.

**Conclusions:**

Compared with IVF, ICSI was associated with a reduction in live births when ART was indicated due to female-only factors. Additionally, no significant improvements were associated with the use of ICSI in cycles with unexplained infertility. Our findings impose a critical reevaluation regarding the use of ICSI over IVF for cases with female-only factors and unexplained infertility.

**Supplementary Information:**

The online version contains supplementary material available at 10.1186/s12967-024-05515-x.

## Background

Assisted reproductive technology (ART) has undergone a profound transformation since the inception of intracytoplasmic sperm injection (ICSI) in 1992 [1].

This technique quickly became an integral component of infertility treatments due to its advantages in addressing male-related infertility challenges, such as altered semen parameters and azoospermia. ICSI is the gold standard for couples with these conditions or when preimplantation genetic testing is required. Moreover, although strong evidence is still lacking, ICSI is empirically used in conditions such as failed fertilization in previous cycles, a low number of available oocytes, or advanced maternal age.

According to data from the European Society of Human Reproduction and Embryology (ESHRE) European IVF Monitoring, since 2002, ICSI has surpassed conventional in vitro insemination (IVF) in the proportion of techniques applied, with ICSI accounting for approximately three-fourths of all in vitro fertilization cycles in Europe [2].

It has been reported that in the US, the percentage of cycles using ICSI increased dramatically even if the percentage of infertility diagnoses attributed to male-factor conditions remained stable, indicating a growing application of ICSI for non-male-factor infertility conditions [3]. This pattern remains consistent globally, as documented in the US, where ICSI rates per clinic varied significantly across geographic regions [4], and worldwide [5], highlighting that the increased use of ICSI did not correlate with a rise in male factor diagnoses. Of note, previous randomized clinical trials consistently failed to demonstrate the advantages of ICSI over IVF in the presence of a non-severe male infertility factor [6–10], and scientific societies have provided recommendations against the routine use of ICSI for all patients undergoing assisted reproductive technologies (ARTs) [11–14].

The continued prevalence of ICSI outside male indications in real-world clinical practice may be rooted in a multitude of factors, including clinical practice patterns, health insurance coverage, patient preferences, and incomplete awareness of outcomes achieved through IVF. On the other hand, this surge raises pertinent questions about the safety and appropriateness of the nearly indiscriminate use of ICSI over IVF in the absence of documented advantages. Moreover, ICSI introduces an additional potentially invasive step in assisted reproduction, and its economic implications remain a subject of significant interest [15].

In the present study, we drew upon real-world data from the Human Fertilization and Embryology Authority (HFEA) registry to gain insight into the utilization of ICSI in contemporary practice. The choice of the HFEA registry data is linked not only to its recognition as one of the largest IVF registries but also to its request to explain the reason for using ICSI in any particular case in the patient’s medical records. This allowed us to assess not only the rates of ICSI utilization compared to those of IVF but also the efficacy of the two approaches based on different causes of couple infertility. A previous analysis of the HFEA dataset, focusing mainly on cycles with poor ovarian response, suggested that ICSI does not confer any benefit in improving the live birth rate compared to IVF [7]. Our current study evaluated whether the preferred use of ICSI over IVF as a fertilization technique in couples without male factor infertility has an influence on clinical outcomes.

## Methods

### Study design

This was a retrospective case‒control study of ART cycles performed in the UK from 2005 to 2018. The data were obtained from the freely available HFEA database (https://www.hfea.gov.uk/about-us/data-research/).

### Participants

To construct our dataset, we extracted cycles that met specific criteria among those available in the HFEA registry. These criteria included being the patient’s first ART cycle and having complete information regarding the following key variables: cause of infertility treatment (indication), female partner’s age, number of oocytes retrieved, a clear and exclusive allocation to ICSI or IVF, and live birth occurrence.

As our study exclusively relied on publicly available registry data, ethical approval was not sought in accordance with Title 45 of the Code of Federal Regulations, Part 46 (45 CFR §46). The HFEA ensures stringent measures for data de-identification before making it accessible to the public. Consequently, patient privacy and confidentiality were maintained in line with the highest ethical standards.

**Procedures and outcomes:**

Data were extracted by two authors (AP, AV) in November 2023. The causes of infertility were simplified into three groups: (1) female (females with only endometriosis, tubal disease, ovulatory disorders, or cervical factors); (2) male (those with only male infertility or with both male and female infertility); and (3) unexplained (those without specific causes of infertility). The selection process ensured that our analysis was based on comprehensive data. The following exclusion criteria were used to filter out cycles from the dataset: entries indicating previous ART cycles, no oocytes retrieved, frozen cycles, donated embryos/sperm or oocytes, reason for performing the cycle other than “treatment now” (the current ART cycle is being undertaken with the immediate goal of achieving a pregnancy), use of both IVF and ICSI in the same cycle, surrogacy, missing information in key variables, PGT cycles, and presence of incongruencies such as number of obtained embryos > number of inseminated oocytes. Cycles with a male factor were excluded from further analysis.

The obtained dataset retained variables that were used to compute other variables, as reported in Supplementary Table 1, and was analysed with two main approaches: binomial logistic regression analysis and matched pair analysis.

As a primary outcome measure, the effectiveness of ICSI compared to that of IVF in achieving live births within the first fresh cycle was evaluated. For available data in the registry, a fresh cycle can end with the following principal outcomes: (1) no embryo transfer for no available embryos; (2) no embryo transfer for freezing of all of the available embryos (freeze-all strategy); (3) embryo transfer without a live birth; and (4) embryo transfer with a live birth. This metric was chosen due to the absence of information on cumulative live births in the public HFEA registry. The secondary outcomes were the absence of viable embryos or the rate of cycle cancellation, which was used as a surrogate measure for total fertilization failure occurrence; the implantation rate, which is defined as the number of fetal sacs with heart pulsation per number of embryos transferred; the miscarriage rate; and the main neonatal outcomes, including the secondary sex ratio (reported as the number of males per 100 females at birth). Since the HFEA dataset for 2017-2018 reports the number of inseminated oocytes or obtained embryos as a categorical variable only, the fertilization rate was computed only for cycles performed between 2005 and 2016. The main neonatal outcomes included weeks of gestation, birthweight, and male/female ratio and were limited to pregnancies starting with a single foetus.

In calculating the implantation rate, cases where a single embryo transfer resulted in the development of twins due to embryo splitting were treated as a single implantation event. To facilitate this and ensure a precise calculation of the implantation rate per transferred embryo, a distinct dataset was created from the main dataset where each transferred embryo was  represented as an individual entry.

An additional dataset was derived from the main dataset using matched-pair data extraction. In this approach, couples treated with IVF were matched at a 1:1 ratio to couples treated with ICSI based on belonging to the same category for all the following key variables: indication for infertility treatment (female or unexplained factor), female age category (18–34, 35–37, 38–39, 40–42, 43–44, 45–50 years), and the number of oocytes allocated to IVF or ICSI (1–5, 6–10, 11–15, 16–20, 21–25, 26–30, 31–35, 36–40, > 40 oocytes). Moreover, couples were matched according to year of treatment (± 2 years). This method allowed us to reasonably isolate the specific impact of ICSI versus IVF on live birth rates and secondary outcomes within comparable groups. The same outcomes as those in the entire cohort of cycles were evaluated, and the results are presented based on 2 distinct categories indicating the simplified causes of infertility.

### Statistical analysis:

**Fig. 1:**
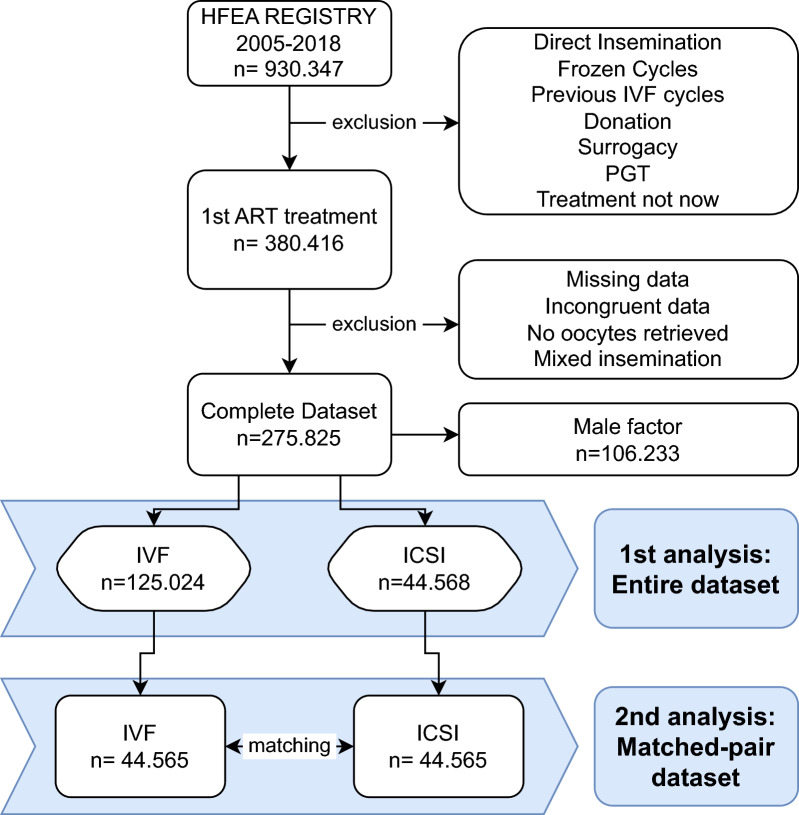
Process of selection and analysis of the study. IVF: conventional in vitro insemination; PGT: preimplantation genetic testing; ART: assisted reproductive technology; HFEA: Human Fertilisation and Embryology Authority

The sample size determination involved selecting cycles from the HFEA registry, starting from the most recent release and working backwards until reaching the calculated sample size. This calculation was based on specific assumptions: (1) a 3:1 allocation ratio of IVF to ICSI treatments in cycles without a male cause of infertility in the HFEA registry; (2) a 30% live birth rate with IVF; and (3) a 1% type I error, and an 80% study power to detect a 1% difference between IVF and ICSI in the first fresh cycles without a male cause of infertility. Based on these assumptions, a total of 160,000 cycles were selected for inclusion, with 120,000 cycles in the IVF group and 40,000 cycles in the ICSI group. We estimated that a subgroup analysis based on a specific indication, such as female-only factor, with a minimum of 60,000 total cycles, would have maintained 80% power to detect a 1.5% variation in the main outcome, which was deemed suitable for the study’s aims. Ultimately, the requested sample size was obtained considering datasets released for the following periods: 2017–2018, 2015–2016, 2010–2014, and 2005–2009. The detailed selection process is depicted in Fig. 1.

Categorical variables were described as percentages. The confidence intervals (95% CI) for the outcomes reported as rates were computed utilizing a binomial distribution, and differences between ICSI and IVF groups were assessed through the chi-square test. Continuous variables were  reported as the mean and standard deviation (SD) and were compared with the t test for independent samples or as the median and interquartile range and compared with the Kruskal‒Wallis test. The analysis of the datasets was performed using a binary logistic regression model when the independent variable was dichotomous (live birth, cycle cancellation). The following basal characteristics that were significantly different between IVF and ICSI groups were used as covariates in the logistic regression model: female age (category), number of oocytes allocated to insemination (category), number of embryos transferred, stage of development of transferred embryos (cleavage, blastocyst stage or other), type of female cause of infertility (endometriosis, ovulatory disorders, tubal factor), and year of treatment. To reduce redundancy and correlation in predictors, we utilized two combined variables obtained via principal component factor analysis, namely, female age, the number of inseminated oocytes (PCA1) and the number and stage of transferred embryos (PCA2). PCA1 was used in the regression models aimed at evaluating the number of live births per cycle and cycle cancellation rate. PCA2 was added for the miscarriage rate per clinical pregnancy, implantation rate and male/female ratio in newborns. The analysis of implantation was conducted on a specific dataset utilizing generalized estimating equations. This approach took into account the correlation among embryos originating from the same woman and transferred together, addressing the interdependence in our analysis. The results were reported according to the cause of infertility using odds ratios (ORs) and adjusted odds ratios (aORs) with 95% CIs. The confidence interval of the secondary sex ratio (SSR) was computed using the Koopman asymptotic method [16]. Statistical analysis was performed with SPSS software ver. 20 (IBM SPSS Statistics for Windows, Version 20.0. Armonk, NY: IBM Corp), and pair matching was performed with MedCalc software ver. 19.7 (MedCalc Software, Ostend, Belgium).

## Results

The final dataset comprised 275,825 first ART cycles from the HFEA registry spanning the years 2005-2018 with complete data on essential variables together with the main clinical outcomes. The basal characteristics of the patients included in the dataset are reported in Table 1, and the selection process is summarized in Figure 1. Throughout the considered period, there was a consistent balance in treatment distribution, with equal proportions of cycles allocated to IVF (50.0%) and ICSI (50.0%); however, a slight but significant increase in the proportion of ICSI cycles compared to that of IVF cycles was observed in recent years (48% versus 52% in 2005–2009 and 51% versus 49% in 2015-2018, p < 0.001). Although not used in the present report, 90% of cycles with male factor infertility and 80% of cycles with mixed infertility factors utilized ICSI. After excluding cycles with a male cause of infertility, 125,024 out of 169,592 couples underwent IVF in their initial cycle, comprising 79% of cycles with female-only infertility and 70% of cycles with unexplained infertility (p < 0.001). The rates of main female infertility causes were 6.8% endometriosis, 12.7% ovulatory disorders and 14.9% tubal factors in the entire dataset; these 3 groups of female factors were preferentially treated with IVF (p < 0.001). The main clinical and embryological outcomes of the included fresh cycles were compared between IVF and ICSI according to the main cause of infertility and are summarized in Table 2. Overall, no significant differences were observed in live birth rate between IVF (28.5%) and ICSI (28.1%) (p = 0.052). In couples with female-only causes of infertility, IVF treatment was associated with a significantly greater percentage of live births (29.7%) than ICSI (28.8%) (p = 0.039), while no differences were observed in couples with unexplained infertility.

**Table 1:** Main characteristics of fresh first cycles treated with IVF or ICSI by cause of infertility, HFEA registry

**Characteristic**	**Overall**			**Female factor only**			**Unexplained**			**Male factor**		
	**IVF n = 137,969**	**ICSI n = 137,856**	**p**	**IVF n = 53,374**	**ICSI n = 13,995**	**p**	**IVF n = 71,650**	**ICSI n = 30,573**	**p**	**cIVF n = 12,945**	**ICSI n = 93,288**	**p**
Year of treatment			< 0.001			< 0.001			< 0.001			< 0.001
2005–2009	44,198 (32.0%)	41,160 (29.9%)		19,344 (36.2%)	4155 (29.7%)		20,150 (28.1%)	6858 (22.4%)		4704 (36.3%)	30,147 (32.3%)	
2010–2014	51,516 (37.3%)	52,894 (38.4%)		19,884 (37.3%)	5392 (38.5%)		26,993 (37.7%)	11,817 (38.7%)		4639 (35.8%)	35,685 (38.3%)	
2015–2018	42,255 (30.6%)	43,802 (31.8%)		14,146 (26.5%)	4448 (31.8%)		24,507 (34.2%)	11,898 (38.9%)		3602 (27.8%)	27,456 (29.4%)	
Female age (years)			< 0.001			< 0.001			< 0.001			< 0.001
18–34	65,237 (47.3%)	76,249 (55.3%)		29,998 (56.2%)	7876 (56.3%)		28,907 (40.3%)	13,326 (43.6%)		6332 (48.9%)	55,047 (59.0%)	
35–37	32,892 (23.8%)	29,356 (21.3%)		12,131 (22.7%)	2952 (21.1%)		17,696 (24.7%)	6965 (22.8%)		3065 (23.7%)	19,439 (20.8%)	
38–39	20,549 (14.9%)	16,873 (12.2%)		6494 (12.2%)	1662 (11.9%)		12,189 (17.0%)	4693 (15.4%)		1866 (14.4%)	10,518 (11.3%)	
40–42	15,068 (10.9%)	11,894 (8.6%)		3876 (7.3%)	1180 (8.4%)		9838 (13.7%)	4001 (13.1%)		1354 (10.5%)	6713 (7.2%)	
43–44	3394 (2.5%)	2687 (1.9%)		709 (1.3%)	240 (1.7%)		2412 (3.4%)	1194 (3.9%)		273 (2.1%)	1253 (1.3%)	
45–50	829 (0.6%)	797 (0.6%)		166 (0.3%)	85 (0.6%)		608 (0.8%)	394 (1.3%)		55 (0.4%)	318 (0.3%)	
Cause of infertility			< 0.001			< 0.001			< 0.001			< 0.001
Female	53,374 (79.2%)	13,995 (20.8%)		53,374 (79.2%)	13,995 (20.8%)		0 (0%)	0 (0%)		0 (0%)	0 (0%)	
Male	8734 (10.2%)	77,004 (89.8%)		0 (0%)	0 (0%)		0 (0%)	0 (0%)		8734 (10.2%)	77,004 (89.8%)	
Female and male	4211 (20.5%)	16,284 (79.5%)		0 (0%)	0 (0%)		0 (0%)	0 (0%)		4211 (20.5%)	16,284 (79.5%)	
Unexplained	71,650 (70.1%)	30,573 (29.9%)		0 (0%)	0 (0%)		71,650 (70.1%)	30,573 (29.9%)		0 (0%)	0 (0%)	
N° of oocytes collected			< 0.001			0.03			< 0.001			< 0.001
1–5	35,541 (25.8%)	30,523 (22.1%)		12,373 (23.2%)	3237 (23.1%)		19,876 (27.7%)	8221 (26.9%)		3292 (25.4%)	19,065 (20.4%)	
6–10	47,672 (34.6%)	47,969 (34.8%)		17,730 (33.2%)	4695 (33.5%)		25,355 (35.4%)	10,801 (35.3%)		4587 (35.4%)	32,473 (34.8%)	
11–15	31,139 (22.6%)	33,245 (24.1%)		12,579 (23.6%)	3150 (22.5%)		15,660 (21.9%)	6671 (21.8%)		2900 (22.4%)	23,424 (25.1%)	
16–20	14,446 (10.5%)	16,001 (11.6%)		6267 (11.7%)	1643 (11.7%)		6831 (9.5%)	3004 (9.8%)		1348 (10.4%)	11,354 (12.2%)	
21–25	5696 (4.1%)	6332 (4.6%)		2688 (5.0%)	752 (5.4%)		2515 (3.5%)	1220 (4.0%)		493 (3.8%)	4360 (4.7%)	
26–30	2179 (1.6%)	2363 (1.7%)		1037 (1.9%)	304 (2.2%)		936 (1.3%)	412 (1.3%)		206 (1.6%)	1647 (1.8%)	
31–35	797 (0.6%)	902 (0.7%)		428 (0.8%)	142 (1.0%)		302 (0.4%)	151 (0.5%)		67 (0.5%)	609 (0.7%)	
36–40	316 (0.2%)	342 (0.2%)		164 (0.3%)	44 (0.3%)		121 (0.2%)	53 (0.2%)		31 (0.2%)	245 (0.3%)	
> 40	183 (0.1%)	179 (0.1%)		108 (0.2%)	28 (0.2%)		54 (0.1%)	40 (0.1%)		21 (0.2%)	111 (0.1%)	

Data are reported as Number and percentage per column

IVF,  conventional in vitro insemination; ICSI, intracytoplasmic sperm injection

**Table 2:** Outcomes of cycles according to the cause of infertility

**Outcome**		**Female factor**			**Unexplained factor**		
		**IVF**	**ICSI**	**p**	**IVF**	**ICSI**	**p**
Cycles	N	53,374	13,995		71,650	30,573	
Fertilization rate*	Median (IQR)	67% (50–83%)	75% (57–88%)	< 0.001	67% (47–83%)	73% (54–88%)	< 0.001
Freeze all	N (%)	2942 (5.5%)	821 (5.9%)	0.10	2894 (4.0%)	1384 (4.5%)	< 0.001
No available embryos	N (%)	2741 (5.1%)	561 (4.0%)	< 0.001	5083 (7.1%)	1587 (5.2%)	< 0.001
N of embryos transferred	Mean (SD)	1.56 (0.53)	1.59 (0.55)	< 0.001	1.58 (0.55)	1.59 (0.57)	0.050
1	N (%)	21,539 (45.2%)	5570 (44.2%)	< 0.001	28,303 (44.5%)	12,438 (45.1%)	< 0.001
2	N (%)	25,451 (53.4%)	6696 (53.1%)		33,597 (52.8%)	14,007 (50.7%)	
3	N (%)	701 (1.5%)	347 (2.8%)		1773** (2.8%)	1157 (4.2%)	
Embryo transfer stage							
Cleavage stage	N (%)	28,901 (60.6%)	7162 (56.8%)	< 0.001	37,686 (59.2%)	15,180 (55.0%)	< 0.001
Blastocyst stage	N (%)	18,121 (38.0%)	5219 (41.4%)		25,188 (39.6%)	11,903 (43.1%)	
Other/unspecified	N (%)	669 (1.4%)	232 (1.8%)		799 (1.3%)	519 (1.9%)	
Cycles with ≥ 1 embryo stored	N (%)	22,562 (42.3%)	5555 (39.7%)	< 0.001	27,157 (37.9%)	11,348 (37.1%)	0.018
1–5 embryos	N (%)	16,927 (31.7%)	4398 (31.4%)	< 0.001	21,668 (30.2%)	9449 (30.9%)	< 0.001
> 5 embryos	N (%)	5635 (10.6%)	1157 (8.3%)		5489 (7.7%)	1899 (6.2%)	
Pregnancies with FHB	N; % (95%CI)	17,893; 33.5% (33.1–33.9%)	4617; 33.0% (32.2–33.8%)	0.23	22,669; 31.6% (31.3–32.0%)	9743; 31.9% (31.4–32.4%)	0.43
Implantation rate (with FHB)							
Single embryo transfer	N Implanted/transferred	8331/21539	2057/5570	0.017	10,392/28303	4462/12438	0.10
	% (95%CI)	38.7% (38.0–39.3%)	36.9% (35.7–38.2%)		36.7% (36.2–37.3%)	35.9% (35.0–36.7%)	
Double embryo transfer	N Implanted/transferred	12,363/50902	3288/13392	0.53	15,270/67194	6379/28014	0.88
	% (95%CI)	24.3% (23.9–24.7%)	24.6% (23.8–25.3%)		22.7% (22.4–23.0%)	22.8% (22.3–23.3%)	
Triple embryo transfer	N Implanted/transferred	164/2103	70/1041	0.24	435/5320**	312/3471	0.19
	% (95%CI)	7.8% (6.7–8.9%)	6.7% (5.2–8.2%)		8.2% (7.4–8.9%)	9.0% (8.0–9.9%)	
Live birth occurrence	N; % (95%CI)	15,847; 29.7% (29.3–30.1%)	4030; 28.8% (28.0–29.5%)	0.039	19,847; 27.7% (27.4–28.0%)	8479; 27.7% (27.2–28.2%)	0.91

^*^cycles perfomed in 2017–2018 were excluded since the number of inseminated/fertilized eggs are available as categorical data only; ** includes 1 transfer with 4 embryos

IVF, conventional in vitro insemination; ICSI, intracytoplasmic sperm injection, FHB, fetal heartbeat; IQR, interquartile range; SD, standard deviation; 95% CI, 95% confidence interval

The paired analysis included 44565 IVF and 44565 ICSI cycles matched based on female age, number of inseminated oocytes and indication for fertility treatments. In the matched dataset, IVF and ICSI treatments were distributed across female age groups as follows: 18–34 years (47.4%), 35–37 years (21.5%), 38–39 years (13.9%), 40–42 years (12.1%), 43–44 years (3.5%), and 45–50 years (1.6%). The distribution of the number of inseminated oocytes was as follows: 1–5 (34.5%), 6–10 (35.5%), 11–15 (18.3%), 16–20 (7.5%), 21–25 (2.7%), 26–30 (1.0%), 31–35 (0.3%), 36–40 (0.1%), and more than 40 oocytes (< 0.1%). The main outcomes according to the cause of infertility in the matched pair analysis are reported in Table 3.

**Table 3:** Matched-Pair dataset: outcomes of treatments according to the cause of infertility

**Outcome**		**Female factor**			**Unexplained factor**		
		**IVF**	**ICSI**	**p**	**IVF**	**ICSI**	**p**
Cycles	N	13,992	13,992		30,573	30,573	
Fertilization rate*	Median (IQR)	67% (50–83%)	75% (57–88%)	< 0.001	67% (44–83%)	73% (54–88%)	< 0.001
Freeze all	N (%)	520 (3.7%)	821 (5.9%)	< 0.001	882 (2.9%)	1384 (4.5%)	< 0.001
No available embryos	N (%)	865 (6.2%)	561 (4.0%)	< 0.001	2335 (7.6%)	1587 (5.2%)	< 0.001
N of embryos transferred	Mean (SD)	1.59 (0.52)	1.59 (0.55)	0.40	1.60 (0.54)	1.59 (0.57)	0.33
1	N (%)	5343 (38.2%)	5568 (39.8%)	< 0.001	11,683 (38.2%)	12,438 (40.7%)	< 0.001
2	N (%)	7070 (50.5%)	6695 (47.8%)		14,737 (48.2%)	14,007 (45.8%)	
3	N (%)	194 (1.4%)	347 (2.5%)		718 (2.3%)	1157 (3.8%)	
Embryo transfer stage							
Cleavage stage	N (%)	8436 (66.9%)	7160 (56.8%)	< 0.001	17,710 (65.3%)	15,180 (55.0%)	< 0.001
Blastocyst stage	N (%)	4006 (31.8%)	5222 (41.4%)		9099 (33.5%)	11,911 (43.2%)	
Other/unspecified	N (%)	165 (1.3%)	228 (1.8%)		329 (1.2%)	511 (1.9%)	
Cycles with ≥ 1 embryo stored	N (%)	5064 (36.2%)	5554 (39.7%)	< 0.001	9654 (31.6%)	11,348 (37.1%)	< 0.001
1–5 embryos	N (%)	4192 (30.0%)	4397 (31.4%)	< 0.001	8102 (26.5%)	9449 (30.9%)	< 0.001
> 5 embryos	N (%)	872 (6.2%)	1157 (8.3%)		1152 (5.1%)	1899 (6.2%)	
Pregnancies with FHB	N; % (95%CI)	4479; 32.0% (31.2–32.8%)	4616; 33.0% (32.2–33.8%)	0.08	9303; 30.4% (29.9–31.0%)	9743; 31.9% (31.4–32.4%)	< 0.001
Implantation rate (with FHB)							
Single embryo transfer	N Implanted/transferred	1933/5343	2057/5568	0.41	4044/11683	4462/12438	0.041
	% (95%CI)	36.2% (24.9–37.5%)	36.9 (35.7–38.2%)		34.6% (33.8–35.5%)	35.9% (35.0–36.7%)	
Double embryo transfer	N Implanted/transferred	3299/14140	3287/13390	0.018	6561/29474	6379/28014	0.14
	% (95%CI)	23.3% (22.6–24.0%)	24.5% (23.8–25.3%)		22.3% (21.8–22.7%)	22.8% (22.3–23.3%)	
Triple embryo transfer	N Implanted/transferred	33/582	70/1041	0.40	166/2154	312/3471	0.09
	% (95%CI)	6.0% (4.1–7.9%)	6.7% (5.2–8.2%)		7.7% (6.6–8.8%)	9.0% (8.0–9.9%)	
Live Birth Occurrence	N; % (95%CI)	3992; 28.5% (27.8–29.3%)	4029; 28.8% (28.1–29.6%)	0.93	8120; 26.6% (26.1–27.1%)	8479; 27.7% (27.2–28.2%)	0.001

^*^cycles perfomed in 2017–2018 were excluded since the number of inseminated/fertilized oocytes are available as categorical data only

IVF,  conventional in vitro insemination; ICSI, intracytoplasmic sperm injection; FHB, fetal heartbeat; IQR, interquartile range; SD, standard deviation; 95%CI, 95% confidence interval

The crude paired analysis showed no significant differences between IVF and ICSI for live birth rate in female factors and a statistically significant increase in live birth rate using ICSI in couples with unexplained infertility (Table 3). As shown in Table 4, after adjustment for confounding factors, the odds of having a live birth in cycles with female-only infertility decreased by factors of 0.95 (95% CI 0.91–0.99) in total cycles and 0.91 (95% CI 0.86–0.96) in matched pair analysis with the use of ICSI compared to IVF. No significant differences emerged in the unexplained factor cycles.

**Table 4:** Odds Ratios for main outcomes according to the cause of infertility in ICSI compared to IVF cycles

	Female factor		Unexplained factor	
	Entire dataset	Matched-pair dataset	Entire dataset	Matched-pair dataset
Live birth/cycle				
Crude OR	0.96 (0.92–1.00)	1.01 (0.96–1.07)	1.00 (0.97–1.03)	1.06 (1.02–1.10)
Adjusted OR*	0.95 (0.91–0.99)	0.91 (0.86–0.96)	0.98 (0.95–1.01)	0.97 (0.93–1.01)
Cycle cancellation				
Crude OR	0.77 (0.70–0.85)	0.63 (0.57–0.71)	0.60 (0.55–0.64)	0.60 (0.56–0.64)
Adjusted OR*	0.72 (0.65–0.79)	0.66 (0.59–0.74)	0.72 (0.68–0.76)	0.64 (0.60–0.68)
Implantation				
Crude OR	0.96 (0.92–0.99)	1.04 (1.00–1.10)	0.97 (0.95–1.00)	1.03 (1.00–1.06)
Adjusted OR**	0.92 (0.89–0.96)	0.92 (0.88–0.97)	0.93 (0.91–0.96)	0.93 (0.90–0.96)
Miscarriage				
Crude OR	1.13 (1.02–1.24)	1.19 (1.05–1.35)	1.05 (0.98–1.13)	1.03 (0.95–1.12)
Adjusted OR**	1.12 (1.01–1.23)	1.24 (1.09–1.41)	1.08 (1.01–1.16)	1.09 (1.00–1.19)
Male sex in newborns (in single pregnancies)				
Crude OR	0.84 (0.77–0.91)	0.86 (0.77–0.96)	0.86 (0.81–0.92)	0.88 (0.82–0.95)
Adjusted OR**	0.83 (0.77–0.91)	0.86 (0.77–0.96)	0.87 (0.81–0.92)	0.88 (0.81–0.95)

OR, Odds Ratio with 95% Confidence Intervals in brackets; IVF, conventional in vitro insemination; ICSI, intracytoplasmic sperm injection; Statistically significant results are indicated by confidence interval ranges that do not include the value 1 (p < 0.05)

^*^Adjustment was performed including PCA1 (principal component analysis based on female age and number of inseminated oocytes), type of female factor, year of treatment

^**^Adjustment was performed including PCA1 (principal component analysis based on female age, number of inseminated oocytes), PCA2 (number and stage of transferred embryos), type of female factor, year of treatment

In the whole cohort of cycles with female-only or unexplained infertility, IVF cycles showed a significantly higher cancellation rate (6.3% with IVF versus 4.8% in ICSI, p < 0.001) due to the absence of viable embryos to be transferred or cryopreserved. This led to an overall crude odds of total fertilization failure being significantly increased by a factor of 1.32 (95% CI 1.26–1.38).

Logistic regression analysis of the entire and matched-pair datasets confirmed a greater percentage of cycles resulting in cancellation due to the absence of viable embryos in the IVF group than in the ICSI group, independent of the cause of infertility, with an adjusted odds ratio ranging between 1.39 and 1.56 (Table 4).

The implantation rates, in women receiving 2, or 3 embryos, were similar between the IVF and ICSI groups. However, in cycles where only one embryo was transferred, the implantation rate was significantly greater with IVF (38.7% versus 36.9%, p < 0.05) (Table 2). The matched-pair analysis of cases with female-only factors revealed a significantly higher implantation rate with double embryo transfers in ICSI cycles, whereas cases with unexplained infertility showed a significantly higher implantation rate with single embryo transfer in ICSI cycles (Table 3). However, after adjustment for PCA1 (female age, number of inseminated oocytes), PCA2 (number of embryos transferred, stage of embryo transfer: cleavage stage, blastocyst stage or other), female infertility factor, and year of treatment, IVF was associated with an increased chance of implantation in couples with both female-only and unexplained infertility factors compared to ICSI (Table 4).

The miscarriage rate was greater for ICSI (13.0%) than for IVF (12.0%) in the whole cohort (p = 0.004), with an odds ratio equal to 1.09 (95% CI 1.03–1.15). After the results were split according to the infertility factor, the difference remained statistically significant, particularly in cycles with female infertility (Tables 5 and 6). The adjusted odds ratios for miscarriage confirmed this tendency (Table 4). When examining the obstetric outcomes according to treatment indications, both IVF and ICSI yielded comparable results concerning gestational weeks and birthweight, as illustrated in Table 5 for the entire dataset and in Table 6 for the matched-pair analysis considering only single live births originating from pregnancies starting with a single fetal heartbeat. Minimal differences observed in the distribution of neonatal birthweight are likely attributable to varying rates of missing values within the groups. The sex of newborns was significantly associated with insemination technique: overall, among 32,505 newborns from cycles with female or unexplained infertility and with complete information from single pregnancies, 52.8% were males in the IVF group and 48.9% were males in the ICSI group, (p < 0.001). Specifically, in the female-only infertility group treated with IVF, 5683 out of 10,755 newborns (52.8%) were males, whereas with ICSI treatment, 1290 out of 2665 newborns (48.4%) were males (p < 0.001). In the unexplained infertility group, 7077 out of 13,410 newborns (52.8%) from IVF were males, whereas the rate of males with ICSI treatment was 2789 out of 5675 newborns (49.1%) (p < 0.001).

**Table 5:** Outcomes of pregnancies in IVF and ICSI groups according to the cause of infertility

**Outcome**		Female factor			Unexplained factor		
		IVF	ICSI	p	IVF	ICSI	p
Among pregnancies with FHB	N	17,893	4617		22,669	9743	
Fetal sacs with FHB							
1	N (%)	14,804 (82.7%)	3800 (82.3%)	0.84	19,085 (84.2%)	8267 (84.9%)	0.32
2	N (%)	3027 (16.9%)	799 (17.3%)		3494 (15.4%)	1442 (14.8%)	
3–4	N (%)	62 (0.3%)	18 (0.4%)		90 (0.4%)	34 (0.3%)	
Miscarriage	N (%)	2055 (11.5%)	589 (12.8%)	0.017	2832 (12.5%)	1274 (13.1%)	0.15
Live births	N	15,847	4030		19,847	8479	
Multiple live births	N (%)	2509 (15.8%)	674 (16.7%)	0.33	2861 (14.4%)	1189 (14.0%)	0.76
Among single live births starting with 1 FHB	N	12,943	3266		16,440	7070	
Weeks of gestation				0.45			0.39
< 32	N (%)	271 (2.1%)	56 (1.7%)		286 (1.7%)	105 (1.7%)	
32–37	N (%)	2079 (16.1%)	544 (16.7%)		2368 (14.4%)	1048 (14.8%)	
≥ 38	N (%)	10,502 (81.1%)	2642 (80.9%)		13,675 (83.2%)	5875 (83.1%)	
Missing data	N (%)	91 (0.7%)	24 (0.7%)		111 (0.7%)	42 (0.6%)	
Birth weight (g)				0.22			0.037
< 1500	N (%)	218 (1.7%)	46 (1.4%)		193 (1.2%)	72 (1.0%)	
1500–2499	N (%)	804 (6.2%)	204 (6.2%)		980 (6.0%)	386 (5.5%)	
2500–3999	N (%)	8844 (68.3%)	2196 (67.2%)		11,079 (67.4%)	4756 (67.3%)	
≥ 4000	N (%)	797 (6.2%)	192 (5.9%)		1052 (6.4%)	413 (5.8%)	
Missing data	N (%)	2280 (17.6%)	628 (19.2%)		3136 (19.1%)	1433 (20.4%)	

FHB = Fetal Heartbeat, including cases of embryo splitting; IVF = conventional in vitro insemination; ICSI = intracytoplasmic sperm injection; 95%CI: 95% confidence interval

**Table 6:** Matched-Pair dataset, outcomes of pregnancies in IVF and ICSI groups according to the cause of infertility

Outcome		Female factor			Unexplained factor		
		IVF	ICSI	p	IVF	ICSI	p
Among pregnanies with FHB	N	4479	4616		9303	9743	
Fetal sacs with FHB				0.84			0.034
1	N (%)	3664 (81.8%)	3799 (82.3%)		7778 (83.6%)	8267 (84.9%)	
2	N (%)	798 (17.8%)	799 (17.33%)		1491 (16.0%)	1442 (14.8%)	
3–4	N (%)	17 (0.3%)	18 (0.4%)		34 (0.3%)	34 (0.3%)	
Miscarriage	N (%)	490 (10.9%)	589 (12.8%)	0.007	1187 (12.8%)	1274 (13.1%)	0.52
Live births	N	3989	4027		8116	8469	
Multiple live births	N (%)	667 (16.7%)	674 (16.7%)	0.98	1214 (15.0%)	1189 (14.0%)	0.09
Among single live births starting with 1 FHB	N	3214	3265		6690	7070	
Weeks of gestation				0.95			0.72
< 32	N (%)	57 (1.8%)	56 (1.7%)		106 (1.6%)	105 (1.5%)	
32–37	N (%)	518 (16.1%)	544 (16.7%)		955 (14.3%)	1048 (14.8%)	
≥ 38	N (%)	2615 (81.4%)	2641 (80.9%)		5594 (83.6%)	5875 (83.1%)	
Missing data	N (%)	24 (0.7%)	24 (0.7%)		35 (0.5%)	42 (0.6%)	
Birth weight (g)				0.68			< 0.001
< 1.500	N (%)	44 (1.4%)	46 (1.4%)		66 (1.0%)	72 (1.0%)	
1.500–2.499	N (%)	191 (5.9%)	204 (6.2%)		360 (5.4%)	386 (5.5%)	
2.500–3.999	N (%)	2123 (66.1%)	2195 (67.2%)		4235 (63.3%)	4756 (67.3%)	
≥ 4.000	N (%)	191 (5.9%)	192 (5.9%)		420 (6.3%)	413 (5.8%)	
Missing data	N (%)	665 (20.7%)	628 (19.2%)		1609 (24.1%)	1443 (20.4%)	

FHB, Fetal Heartbeat, including cases of embryo splitting; IVF, conventional in vitro insemination; ICSI, intracytoplasmic sperm injection; 95%CI, 95% confidence interval

The SSR in the entire IVF cohort was 1.12 (95% CI 1.10–1.14), while in the entire ICSI cohort, it was 0.96 (95% CI 0.93–0.99). The adjusted OR for male sex in newborns compared to female sex from singleton pregnancies was consistently significantly influenced by the technique of insemination both in cycles with female-only and unexplained infertility factors and ranged between 0.84 and 0.88 when IVF was used as the reference category (Table 4).

## Discussion

The shift towards an increased adoption of ICSI described over the years prompts a critical examination of the factors driving this trend and the results achieved accordingly. Notably, this shift may be attributed to evolving preferences, advancements in technology, or emerging clinical evidence [17]. Our study, aimed at contributing to the understanding of this latter aspect, failed to demonstrate a clinical advantage of ICSI over IVF in first ART cycles without a male infertility factor. Moreover, a significant increase in the chance of live birth was observed in couples with female-only factors treated with IVF.

The HFEA registry shows a consistent balance in treatment distribution maintained over the study period, offering an interesting opportunity for the evaluation of results achievable through ICSI compared to IVF [18]. In fact, national registries with a pronounced preferential allocation to ICSI would offer an a priori biased sample. Similarly, cycles with male indications were excluded from our analysis since a balanced comparison between IVF and ICSI would be undermined by the severity of the male factor itself, which is not discernible since the HFEA registry lacks a clear description of the type and severity of male indications, leading to potential confounding.

Our findings on the effects of IVF and ICSI on the first fresh ART cycle shed light on the interaction of variables known to influence the likelihood of live birth, implantation, cycle cancellation, and miscarriage according to the cause of infertility. Notably, while the adjusted analysis for live birth per cycle failed to highlight an advantage of ICSI compared to IVF in unexplained infertility (adjusted OR =0.98, 95% CI 0.95–1.01 in the entire dataset and adjusted OR = 0.97, 95% CI 0.93–1.01 in the matched-paired dataset), it indicated a decrease with ICSI in cycles with female-only factors (adjusted OR = 0.95, 95% CI 0.91–0.99 in the entire dataset and 0.91, 95% CI 0.86–0.96 in the matched-pair analysis). The matched-pair analysis further corroborated and refined the insights gained from the broader dataset. The observation that differences in live birth rates were primarily observed in cases of female-only factor infertility and not in cases of unexplained infertility is consistent with the idea that unexplained infertility may mask undiagnosed male factors that are not detected through standard male evaluations. This suggests that standard male fertility evaluations may not be sensitive enough to detect subtle male factors contributing to infertility in cases where the female partner's fertility issues are unexplained.

The higher cancellation rates due to the absence of viable embryos in IVF cycles, as supported by previous findings [7, 19], warrant further consideration. Despite consistently significant adjusted odds ratios favoring ICSI (ranging between 1.4 and 1.6), the absolute incidence of cancellation (including fertilization failure and embryo cleavage arrest) remained relatively low with both techniques, ranging from 5.1% to 7.6% with IVF and from 4.0% to 5.2% with ICSI depending on infertility factors. Importantly, this higher cancellation rate did not result in a reduced occurrence of live births with IVF, as indicated by the adjusted analysis, likely due in part to a higher implantation rate with IVF.

Similarly, despite a slight increase in the OR for miscarriage in cycles with unexplained infertility associated with the use of ICSI, the chance of a live birth per cycle was not affected by the insemination technique, which failed to demonstrate a clear advantage of IVF or ICSI for this indication. The unexplained infertility group showed the highest cancellation rate (Table 3), which was lower for ICSI compared to IVF (OR = 0.64, with a 7.6% cancellation rate for IVF, p < 0.001). Based on this, we estimated that more than 40 cycles would need to be treated with unnecessary ICSI to prevent one case of cancellation due to total fertilization failure.

Compared with those from ICSI, embryos derived from IVF showed a greater chance of implantation both in females and in women with unexplained infertility. Although a recent randomized trial failed to highlight a better implantation rate with the use of IVF than with ICSI in couples without a male infertility factor [9], our findings align with a comprehensive analysis by Boulet et al. using data from a large USA registry [8], which showed that ICSI resulted in lower implantation rates than did IVF in fresh cycles with non-male factor infertility (23.0% vs. 25.2%; p < 0.001). The slight disparity in implantation rates (1–2%) suggests that only studies with large sample sizes, which are rarely achievable with a prospective study design, are likely to reveal statistically significant differences between ICSI and IVF.

Our study, confirming previous findings by Dean et al.[20] and Supramaniam et al. [21], revealed an imbalance in male/female sex ratios in live births through IVF or ICSI. In particular, our dataset showed a ratio of 112 male newborns per 100 females using IVF and 96 males per 100 females with ICSI; of note, according to the United Nations registry, the ratio of males to females in the UK in the period 2005–2018 ranged between 104.9 and 105.5 [22], suggesting that in vitro fertilization can significantly alter this ratio, as previously reported and discussed [21]. Our data support a skewed male‒female ratio in newborns between ICSI and IVF, although the direction of the skew differed between the two methods. This observation could indicate that there might be underlying factors influencing the sex ratio of newborns born through these ART procedures. Understanding the reasons behind these differences would likely require further investigation and analysis, potentially involving factors such as sperm selection methods, embryo development dynamics or other biological factors associated with ART procedures. Y-bearing spermatozoa were shown to be slightly lighter and faster but also weaker and shorter-lived than X-bearing spermatozoa [23]. Thus, in IVF, the timing proximity between semen collection and exposure of the oocytes could favor male conceptions. Conversely, these factors are not expected to play a role in ICSI when the embryologist arbitrarily chooses the spermatozoa to inject, determining a primary sex ratio of 1:1 between the two sexes. Our research is not aimed at elucidating the causes, but it is noteworthy that the confirmed skew persisted even after adjusting for key confounding factors such as female age, infertility factors, and stage of embryo transfer, at least in couples without a male infertility factor, emphasizing the need for dedicated investigations into the underlying mechanisms of insemination technique. The arbitrary and unsupported use of ICSI favoring the birth of female newborns compared to the general population could be viewed as an inevitable and acceptable effect when the technique is used for male factor infertility (when the use of ICSI is necessary to ensure success), but one could ethically question this unbalance between sexes when the procedure is not necessary. The inopportune use of ICSI may be viewed as an unjustified interference in the mechanisms of evolution with potential social consequences.

Understanding the clinical significance of the observed differences in outcomes is crucial for informing clinical practice. Our findings suggest that, in certain contexts, one technique may outperform the other. For instance, the higher live birth rate resulting from the use of IVF in women with female-only infertility emphasizes the importance of tailoring treatment strategies to specific patient profiles. Although absolute differences in outcomes may appear to be of low magnitude, it is essential to consider broader implications. ICSI, without providing a clear advantage or even diminishing success rates in cases of female-only factors, entails higher costs, increased time consumption, and potential impacts on laboratory organization. Additionally, possible unwanted side effects, particularly neonatal outcomes, should be critically evaluated, as available data, although generally obtained by comparing ICSI to naturally conceived babies, suggest there might be an increased risk of epigenetic disorders, congenital malformations, chromosomal abnormalities, and subfertility in ICSI children. [24, 25]; of note, the risk of birth defects (including cerebral palsy and terminations for defects at any gestational period) has been also differentially associated with the use of ICSI or IVF, with a heightened risk persisting in the case of ICSI even after adjusting for multiple variables such as parental factors [26]. Although our study was not specifically designed to explore such effects and failed to document clinically relevant differences between ICSI and IVF in terms of prematurity or birth weight, professionals are warranted to remain vigilant and consider emerging evidence in these areas.

Our findings align with and contribute to the existing body of evidence derived from various sources, including large retrospective studies [7, 8, 15, 27, 28], and a meta-analysis of randomized clinical trials [6]. The Cochrane meta-analysis, up to February 2023, included three well-designed randomized controlled trials involving 1539 couples undergoing fertility treatment. These analyses found uncertainty in the effect of ICSI versus IVF on live birth rates (risk ratio = 1.11, 95% CI 0.94–1.30, 2 studies, n = 1124, low-certainty evidence), with the chance of live birth ranging from 30% to 41% for ICSI if assumed to be 32% for IVF. For adverse events such as multiple pregnancy, ectopic pregnancy, pre-eclampsia, and prematurity, there was probably little or no difference between the two techniques, and no study reported on stillbirth. For secondary outcomes, the review found uncertainty in the effect on clinical pregnancy rates (risk ratio = 1.00, 95% CI 0.88–1.13, 3 studies, n = 1539, low-certainty evidence), and found probably little or no difference in viable intrauterine pregnancy rates (risk ratio = 1.00, 95% CI 0.86–1.16, 2 studies, n = 1479, moderate-certainty evidence). Miscarriage rates showed little or no difference between the two techniques.

The certainty of evidence was evaluated as low to moderate due to uncertainties in study methodologies, warranting caution in interpreting results and emphasizing the need for further studies to confirm findings. A large retrospective study based on data on fresh IVF and ICSI cycles reported to the US National Assisted Reproductive Technology Surveillance System during 1996–2012 revealed a significant increase in the use of ICSI in fresh ART cycles in the United States, increasing from 36.4% to 76.2%. The greatest surge was observed in cycles without male factor infertility, although ICSI use was not linked to improved postfertilization reproductive outcomes [8]. Based on the same source of data from the USA, another study based on nearly 47,000 patients [15] highlighted no improvements in cumulative live birth rates with the use of ICSI in patients with non-male infertility (60.9% compared to 64.3% in ICSI and IVF, respectively, p > 0.05). Although the results were not described according to the cause of infertility, the study had the advantage of reporting the cumulative live birth rate, accounting for embryo transfers performed with frozen embryos.

Similarly, a previous large study on the HFEA dataset came to similar conclusions, mainly focusing on cycles with a poor response to ovarian stimulation and non-male factors performed between 1991 and 2016. Specifically, IVF compared with ICSI yielded similar results in terms of live birth in cycles with a low yield of oocytes (adjusted OR = 0.97, 99.5% CI 0.90–1.04). Although a subanalysis of the whole cohort according to the specific cause of infertility was not reported, the study confirmed that ICSI was not associated with improved clinical results in the explored groups, such as those based on the number of oocytes retrieved or the number of previous IVF cycles. Interestingly, an analysis of all patients who underwent their first cycle in the poor ovarian response cohort was performed, and no difference was detected in the live birth outcome for either method of fertilization (aOR = 1.03, 95% CI 0.93–1.14) [7]. This previous study revealed a predilection for ICSI over IVF in successive IVF cycles, confirming the preferential adoption of ICSI to address failures in IVF. In particular, a 38% increase in ICSI utilization (OR 1.38, 99.5% CI 1.31–1.46) in couples undergoing their second treatment cycle compared to those undergoing their first attempt was reported. Although intriguing, this finding underscores a potential selection bias, as the inclusion of repetitive cycles may not adequately control for this confounding factor in the study.

Collectively, available studies have consistently converged on a key message: in the absence of a male factor, ICSI does not seem to offer a clear and significant advantage over IVF treatments. Our present results suggest that in the case of female infertility, ICSI can be detrimental in terms of the live birth rate, at least in the first fresh cycle of fresh embryo transfer.

Acknowledging our study's limitations is important for contextualizing our findings. In general, potential biases intrinsic to the availability and quality of retrospective data obtained from registries and unmeasured confounders may influence the generalizability of the results [29]. More specifically, the definition of a male factor infertility indication poses limitations due to the lack of standardized evaluation criteria: the exclusion of a male factor infertility may vary between institutions according to assessment criteria, leading to inconsistencies in data interpretation and potentially skewed outcomes. Thus, caution should be exercised when drawing conclusions from registry data where detailed information on the male factor or sperm analysis is missing, emphasizing the need for standardized approaches to male fertility evaluation in research and clinical practice [30]. Another specific limitation of our study is the absence of information on cumulative live birth rates and the exact number of available surplus embryos, with only categorized data available from 2016 onwards. Detailed information on stored embryos is crucial for obtaining a comprehensive understanding of the chances of cumulative live births and the overall success trajectory of assisted reproductive techniques. While observed differences in individual cycles may offer some insights, it should be recognized that these differences may not necessarily have a significant differential impact on cumulative live births, emphasizing the need for future studies with more comprehensive data to address this aspect. Finally, a limitation regarding the measure used to assess fertilization should be acknowledged. Our evaluation of total fertilization failure and fertilization rate is based on the number of viable embryos, and as such, it does not account for zygotes that may not develop viable embryos. Similarly, to estimate the occurrence of total fertilization failure, we employed the cancellation rate as a surrogate measure. Although this approach may not be as stringent as directly assessing zygote development, it is worth noting that the impact of zygotes failing to progress to viable embryos is anticipated to be modest. This methodology aligns with previous high-impact studies, such as the one conducted by Boulet et al. [8] supporting its application in the context of our research. Nonetheless, recognizing this limitation is essential, and future investigations may explore more comprehensive methodologies to capture early-stage outcomes of zygote development according to the technique of insemination or specific causes of infertility.

Some strengths collectively enhance the credibility and applicability of the present study, offering a robust perspective on the comparative effectiveness of IVF and ICSI within the examined populations. First, the study design focused on first cycles from the HFEA registry, significantly reducing the allocation bias observed in studies involving repetitive cycles or utilizing data from countries with limited IVF use. This approach also eliminates the potential bias associated with including the same patients across different cycles. Furthermore, the study benefits from a substantial dataset, encompassing nearly 170,000 first ART cycles, providing robust statistical power to detect even subtle differences in the main outcome measures (namely, a 1% difference in the live birth rate). Second, the adoption of a dual analytical approach strengthens the reliability of the study’s findings. Logistic regression analysis of the entire dataset and meticulous matched-pair analysis of nearly 90,000 cycles allowed for a broad overview and ensured a reasonably controlled evaluation, minimizing the impact of the main confounding factors. Third, the study goes beyond a simple comparison of live births and offers various important clinical outcomes, such as cycle cancellation, miscarriage rates and implantation rates. The latter outcome deserves further discussion. The evaluation of implantation rates is often criticized for difficulties linked to objective measurements. Unlike conventional approaches, we systematically presented implantation rates based on the number of embryos transferred, revealing a decreasing chance of implantation with an increased number of embryos likely dependent on the lower quality of embryos or poorer prognostic factors of couples associated with the choice of multiple embryo transfers. To isolate the impact of multiple transfers, we created a specialized dataset, assigning a unique entry for each transferred embryo. This approach facilitated a logistic regression analysis that accounted for the interdependence of implantation chances among embryos from the same couple. This method enhances the reliability of our findings, allowing for a reliable interpretation of factors influencing implantation rates. Finally, stratified analysis based on the cause of infertility allows for the examination of outcomes in distinct patient groups, offering insights into the tailored efficacy of IVF and ICSI across different infertility factors.

Future research may focus on examining the outcomes associated with IVF and ICSI, taking into account advancements in laboratory techniques and long-term effects together with the cost/benefit ratio. Importantly, future studies should assess cumulative results over time, spanning multiple transfers from a single aspiration cycle, to provide a more comprehensive understanding of the comparative effectiveness of IVF and ICSI.

## Conclusions

In conclusion, our study suggests that in the absence of a male factor, the live birth rate per cycle is not significantly improved with the use of ICSI compared with that with IVF, and a reduction in the live birth rate can be associated with the use of ICSI in the presence of female fertility factors, despite a higher rate of cycle cancellation associated with IVF. These findings advocate for a critical approach to treatment selection, highlighting the importance of considering factors beyond cancellation rates when making informed decisions in clinical settings.

### Supplementary Information

Additional file 1.

## Data Availability

We used data freely available on the HFEA registry website as mentioned in the manuscript. The anonymised data collected are available as open data via the HFEA website (https://www.hfea.gov.uk/about-us/data-research/).
