# Supplementary material for: Intracytoplasmic sperm injection versus conventional in vitro insemination in couples with non-male infertility factor in the ‘real-world’ setting: analysis of the HFEA registry
Source: J Transl Med. 2024 Jul 28;22:687. doi: 10.1186/s12967-024-05515-x (PMC11283721; doi:10.1186/s12967-024-05515-x)
Supplement: Supplementary file 1 — Additional file 1. [file 12967_2024_5515_MOESM1_ESM.pdf]

Supplementary Table 1. Variables in the main dataset

Name of variable	Type	Source
Patient Age at Treatment	categorical	HFEA dataset
Cause of Infertility - Tubal disease	categorical	HFEA dataset
Cause of Infertility - Ovulatory Disorder	categorical	HFEA dataset
Cause of Infertility - Male Factor	categorical	HFEA dataset
Cause of Infertility - Patient Unexplained	categorical	HFEA dataset
Cause of Infertility - Endometriosis	categorical	HFEA dataset
Cause of Infertility - Cervical factors	categorical	HFEA dataset
Cause of Infertility - Female Factors	categorical	HFEA dataset
Cause of Infertility - Partner Sperm Concentration	categorical	HFEA dataset
Cause of Infertility - Partner Sperm Morphology	categorical	HFEA dataset
Causes of Infertility - Partner Sperm Motility	categorical	HFEA dataset
Cause of Infertility - Partner Sperm Immunological factors	categorical	HFEA dataset
Specific treatment type	categorical	HFEA dataset
Elective Single Embryo Transfer	categorical	HFEA dataset
Fresh Eggs Collected	scale/categorical	HFEA dataset
Fresh Eggs Stored	scale/categorical	HFEA dataset
Total Eggs Mixed	scale/categorical	HFEA dataset
Eggs Mixed With Partner Sperm	scale/categorical	HFEA dataset
Total Embryos Created	scale	HFEA dataset
Eggs Micro-injected	scale/categorical	HFEA dataset
Embryos from Eggs Micro-injected	scale/categorical	HFEA dataset
Embryos Transfered	scale	HFEA dataset
Embryos Transfered from Eggs Micro-injected	scale	HFEA dataset
Embryos Stored For Use By Patient	categorical	HFEA dataset
Embryos (from Eggs Micro-injected) Stored For Use By Patient	categorical	HFEA dataset
Year of Treatment	scale	HFEA dataset
Live Birth Occurrence	categorical	HFEA dataset
Number of Live Births	scale	HFEA dataset
Early Outcome	categorical	HFEA dataset
Number of foetal sacs with fetal pulsation	scale	HFEA dataset
Heart One Weeks Gestation	scale	HFEA dataset
Heart One Birth Outcome	categorical	HFEA dataset
Heart One Birth Weight	categorical	HFEA dataset
Heart OneSex	categorical	HFEA dataset
Heart Two Weeks Gestation	scale	HFEA dataset
Heart Two Birth Outcome	categorical	HFEA dataset
Heart Two Birth Weight	categorical	HFEA dataset
Heart Two Sex	categorical	HFEA dataset
Heart Three Weeks Gestation	scale	HFEA dataset
Heart Three Birth Outcome	categorical	HFEA dataset
Heart Three Birth Weight	categorical	HFEA dataset
Heart Three Sex	categorical	HFEA dataset
Heart Four Weeks Gestation	scale	HFEA dataset
Heart Four Birth Outcome	categorical	HFEA dataset
Heart Four Birth Weight	categorical	HFEA dataset
Heart Four Sex	categorical	HFEA dataset
Stage of embryo transfer	categorical	computed
Progressive_ID	scale	computed
Indication_semplified	categorical	computed
cIVF_or_ICSI	categorical	computed
Embryo_splitting	categorical	computed
Foetal_sacs_without_splitting	scale	computed
Age_combined_eggs_PCA1	scale	computed
Transfered embryos number combined stage_PCA2	scale	computed
Reason _for_NO_embryo_transfer	categorical	computed
Implantation_Rate	scale	computed
